# Supplementary material for: Pyruvate Dehydrogenase Kinase 1 inhibition mediated oxidative phosphorylation enhancement in cartilage promotes osteoarthritis progression
Source: BMC Musculoskelet Disord. 2023 Jul 20;24:597. doi: 10.1186/s12891-023-06585-6 (PMC10357736; doi:10.1186/s12891-023-06585-6)
Supplement: Supplementary file 1 — Additional file 1. [file 12891_2023_6585_MOESM1_ESM.zip › MF.Gsea.1653627316242/GOMF_OXIDOREDUCTASE_ACTIVITY_ACTING_ON_THE_CH_NH2_GROUP_OF_DONORS.html]

Details for gene set GOMF\_OXIDOREDUCTASE\_ACTIVITY\_ACTING\_ON\_THE\_CH\_NH2\_GROUP\_OF\_DONORS[GSEA]

|  || Dataset | 55457\_collapsed\_to\_symbols.cls.cls #OA\_versus\_normal.cls.cls #OA\_versus\_normal\_repos |
| Phenotype | cls.cls#OA\_versus\_normal\_repos |
| Upregulated in class | OA |
| GeneSet | GOMF\_OXIDOREDUCTASE\_ACTIVITY\_ACTING\_ON\_THE\_CH\_NH2\_GROUP\_OF\_DONORS |
| Enrichment Score (ES) | 0.43609434 |
| Normalized Enrichment Score (NES) | 1.2972939 |
| Nominal p-value | 0.14157303 |
| FDR q-value | 0.40489885 |
| FWER p-Value | 1.0 |
Table: GSEA Results Summary

  

Fig 1: Enrichment plot: GOMF\_OXIDOREDUCTASE\_ACTIVITY\_ACTING\_ON\_THE\_CH\_NH2\_GROUP\_OF\_DONORS      
 Profile of the Running ES Score & Positions of GeneSet Members on the Rank Ordered List

  

| SYMBOL | TITLE | RANK IN GENE LIST | RANK METRIC SCORE | RUNNING ES | CORE ENRICHMENT || 1 | LOX | lysyl oxidase [Source:HGNC Symbol;Acc:HGNC:6664] | 872 | 0.550 | 0.0456 | Yes |
| 2 | GLUD1 | glutamate dehydrogenase 1 [Source:HGNC Symbol;Acc:HGNC:4335] | 1279 | 0.462 | 0.1092 | Yes |
| 3 | LOXL1 | lysyl oxidase like 1 [Source:HGNC Symbol;Acc:HGNC:6665] | 1494 | 0.429 | 0.1810 | Yes |
| 4 | GLUD2 | glutamate dehydrogenase 2 [Source:HGNC Symbol;Acc:HGNC:4336] | 1554 | 0.420 | 0.2629 | Yes |
| 5 | DDO | D-aspartate oxidase [Source:HGNC Symbol;Acc:HGNC:2727] | 1839 | 0.380 | 0.3190 | Yes |
| 6 | AOC1 | amine oxidase copper containing 1 [Source:HGNC Symbol;Acc:HGNC:80] | 1992 | 0.362 | 0.3817 | Yes |
| 7 | MAOB | monoamine oxidase B [Source:HGNC Symbol;Acc:HGNC:6834] | 2192 | 0.339 | 0.4361 | Yes |
| 8 | RNLS | "renalase, FAD dependent amine oxidase [Source:HGNC Symbol;Acc:HGNC:25641]" | 2955 | 0.254 | 0.4293 | No |
| 9 | GLDC | glycine decarboxylase [Source:HGNC Symbol;Acc:HGNC:4313] | 3913 | 0.163 | 0.3887 | No |
| 10 | VCAM1 | vascular cell adhesion molecule 1 [Source:HGNC Symbol;Acc:HGNC:12663] | 5283 | 0.049 | 0.2925 | No |
| 11 | DAO | D-amino acid oxidase [Source:HGNC Symbol;Acc:HGNC:2671] | 6252 | -0.024 | 0.2224 | No |
| 12 | LOXL2 | lysyl oxidase like 2 [Source:HGNC Symbol;Acc:HGNC:6666] | 7675 | -0.133 | 0.1393 | No |
| 13 | MAOA | monoamine oxidase A [Source:HGNC Symbol;Acc:HGNC:6833] | 8271 | -0.177 | 0.1295 | No |
| 14 | AOC3 | amine oxidase copper containing 3 [Source:HGNC Symbol;Acc:HGNC:550] | 9375 | -0.262 | 0.0978 | No |
| 15 | PNPO | pyridoxamine 5'-phosphate oxidase [Source:HGNC Symbol;Acc:HGNC:30260] | 10239 | -0.345 | 0.1019 | No |
| 16 | AOC2 | amine oxidase copper containing 2 [Source:HGNC Symbol;Acc:HGNC:549] | 11550 | -0.508 | 0.1048 | No |
Table: GSEA details [plain text format]

  

Fig 2: GOMF\_OXIDOREDUCTASE\_ACTIVITY\_ACTING\_ON\_THE\_CH\_NH2\_GROUP\_OF\_DONORS      
 Blue-Pink O' Gram in the Space of the Analyzed GeneSet

  

Fig 3: GOMF\_OXIDOREDUCTASE\_ACTIVITY\_ACTING\_ON\_THE\_CH\_NH2\_GROUP\_OF\_DONORS: Random ES distribution      
 Gene set null distribution of ES for **GOMF\_OXIDOREDUCTASE\_ACTIVITY\_ACTING\_ON\_THE\_CH\_NH2\_GROUP\_OF\_DONORS**
